# Supplementary material for: The role of quantitative T1 and T2 mapping for detecting minimal hepatic encephalopathy in chronic hepatic schistosomiasis patients
Source: PLoS One. 2026 Jan 6;21(1):e0339588. doi: 10.1371/journal.pone.0339588 (PMC12774341; doi:10.1371/journal.pone.0339588)
Supplement: S1 Table — (DOCX) [file pone.0339588.s001.docx]

**S1 Table. The clinical and laboratory characteristics and T1 and T2 values in MHE and non-MHE cases**

| Parameters | MHE (N=50) | non-MHE (N=38) | P-value |
| --- | --- | --- | --- |
| Gender |  |  |  |
| Female | 19 (38.0%) | 17 (44.7%) | 0.676 |
| Male | 31 (62.0%) | 21 (55.3%) |  |
| Education |  |  |  |
| Above primary school | 5 (10.0%) | 4 (10.5%) | 1.000 |
| Below primary school | 45 (90.0%) | 34 (89.5%) |  |
| Age (y) |  |  |  |
| Mean±SD | 70.7±6.59 | 69.3±7.99 | 0.365 |
| Median [Min, Max] | 71.0 [50.0, 84.0] | 69.0 [47.0, 84.0] |  |
| NCT-A |  |  |  |
| Mean±SD | 57.7±16.4 | 37.7±9.38 | <0.001 |
| Median [Min, Max] | 57.5 [22.0, 95.0] | 38.5 [6.00, 51.0] |  |
| DST |  |  |  |
| Mean±SD | 34.3±13.8 | 45.4±13.5 | <0.001 |
| Median [Min, Max] | 33.5 [11.0, 88.0] | 46.0 [19.0, 75.0] |  |
| AST (U/L) |  |  |  |
| Mean±SD | 46.9±22.6 | 37.7±19.4 | 0.043 |
| Median [Min, Max] | 40.5 [20.0, 117] | 38.0 [3.00, 69.0] |  |
| ALT (U/L) |  |  |  |
| Mean±SD | 53.5±29.0 | 50.2±21.7 | 0.550 |
| Median [Min, Max] | 46.0 [20.0, 155] | 49.5 [3.00, 88.0] |  |
| TB (μmol/L) |  |  |  |
| Mean±SD | 29.3±11.0 | 24.6±11.2 | 0.054 |
| Median [Min, Max] | 28.9 [9.80, 63.9] | 23.0 [8.00, 51.7] |  |
| ALB (g/L) |  |  |  |
| Mean±SD | 29.5±4.10 | 28.7±3.87 | 0.350 |
| Median [Min, Max] | 30.0 [21.0, 36.0] | 28.5 [20.0, 37.0] |  |
| PT (s) |  |  |  |
| Mean±SD | 13.5±1.18 | 12.7±1.03 | 0.358 |
| Median [Min, Max] | 12.7 [11.0, 13.9] | 11.7 [11.0, 13.7] |  |
| INR |  |  |  |
| Mean±SD | 1.26±0.218 | 1.25±0.327 | 0.960 |
| Median [Min, Max] | 1.28 [0.850, 1.45] | 1.20 [0.800, 1.49] |  |
| PLT (10^9^/L) |  |  |  |
| Mean±SD | 98.0±41.1 | 117±39.0 | 0.212 |
| Median [Min, Max] | 93.5 [80.0, 268] | 102 [80.0, 249] |  |
| T1 Frontal Lobe |  |  |  |
| Mean±SD | 1130±192 | 1300±257 | 0.001 |
| Median [Min, Max] | 1120 [742, 1760] | 1250 [894, 1970] |  |
| T1 Temporal Lobe |  |  |  |
| Mean±SD | 1690±393 | 1910±302 | 0.003 |
| Median [Min, Max] | 1710 [789, 2400] | 2010 [1410, 2480] |  |
| T1 Occipital Lobe |  |  |  |
| Mean±SD | 1240±305 | 1400±271 | 0.011 |
| Median [Min, Max] | 1220 [750, 2380] | 1330 [1080, 2380] |  |
| T1 Caudate Nucleus |  |  |  |
| Mean±SD | 1390±246 | 1890±349 | <0.001 |
| Median [Min, Max] | 1400 [841, 1850] | 1790 [1310, 2780] |  |
| T1 Globus Pallidus |  |  |  |
| Mean±SD | 1250±324 | 1790±315 | <0.001 |
| Median [Min, Max] | 1300 [576, 2180] | 1720 [1110, 2550] |  |
| T2 Frontal Lobe |  |  |  |
| Mean±SD | 96.9±61.0 | 95.8±70.1 | 0.938 |
| Median [Min, Max] | 86.0 [72.5, 515] | 83.6 [72.5, 515] |  |
| T2 Temporal Lobe |  |  |  |
| Mean±SD | 102±24.7 | 95.8±18.6 | 0.180 |
| Median [Min, Max] | 93.3 [79.9, 193] | 90.3 [83.6, 193] |  |
| T2 Occipital Lobe |  |  |  |
| Mean±SD | 93.9±11.7 | 91.6±5.06 | 0.226 |
| Median [Min, Max] | 90.2 [81.2, 134] | 91.1 [84.6, 106] |  |
| T2 Caudate Nucleus |  |  |  |
| Mean±SD | 79.9±9.39 | 79.1±8.19 | 0.682 |
| Median [Min, Max] | 78.7 [64.6, 105] | 79.7 [61.6, 105] |  |
| T2 Globus Pallidus |  |  |  |
| Mean±SD | 72.0±14.0 | 76.4±14.3 | 0.149 |
| Median [Min, Max] | 68.8 [49.7, 92.9] | 80.8 [47.8, 103] |  |

ALB, albumin; ALT, alanine aminotransferase; AST, aspartate aminotransferase; DST, digit symbol test; INR, international normalized ratio; MHE, minimal hepatic encephalopathy; NCT-A, number connection test-A; PLT, platelet count; PT, prothrombin time; TB, total bilirubin
