# Supplementary material for: The role of quantitative T1 and T2 mapping for detecting minimal hepatic encephalopathy in chronic hepatic schistosomiasis patients
Source: PLoS One. 2026 Jan 6;21(1):e0339588. doi: 10.1371/journal.pone.0339588 (PMC12774341; doi:10.1371/journal.pone.0339588)
Supplement: Attachment — Submitted filename: PONE-D-25-46086 (Reviewers comments RH).docx [file pone.0339588.s002.docx]

**PONE-D-25-46086**

**The role of quantitative T1 and T2 mapping for detecting minimal hepatic encephalopathy in chronic hepatic schistosomiasis patients**

**Reviewer Comments**

**Robel A. Habte**

The authors’ work significantly enhances the diagnostic utility of quantitative T1 and T2 mapping as an accurate and non-invasive biomarker for detecting MHE in CHS patients. The measurement of T1 and T2 values, as well as magnetic susceptibility values, in different brain regions could contribute to the recognition of early neurological symptoms and the monitoring of disease progression in patients with CHS. However, their findings reveal a strong correlation between T1 values and the globus pallidus in CHS patients with MHI, exhibiting higher sensitivity and specificity diagnostic magnetic values. Since the central theme of the research discussion centers on T1 mapping, manganese deposition, and magnetic susceptibility of the globus pallidus in differentiating MHI from non-MHI in CHS patients. The following fundamental points have to be brought to the attention of the authors:

- There is discordance between the title of the manuscript and the findings of the research study. The title highlights equally the relevance and quantitative role of T1 and T2 mapping in detecting minimal hepatic encephalopathy in patients with chronic hepatic schistosomiasis, while 80% of the discussion prominently emphasizes T1 mapping. The introduction of the manuscript lacks background information and a review of previous work on T2 mapping.
- The authors defined the aim of this study as evaluating whether MRI T1 mapping can be used for clinical diagnoses in MHE in CHS patients. The title should be more suitable if it focuses on T1 mapping because the analysis attempts to explore T2 mapping, which yields non-significant outcomes.
- The physiological effects of age on mental function, the cognitive role of psychometric test outcomes, and natural variations in T1 and T2 values with aging should be considered in the methodological aspect and incorporated as part of the discussion section.
- The mean age of the patients was approximately 70, and most were in their 5th, 6th, and 7th decades of life. The context of this research paper and the study's outcomes should be tailored to the quantitative role and diagnostic accuracy of T1 mapping in diagnosing MHE in middle-aged and older CHS patients.
- The key message of this scientific study is that MRI findings characterized T1 hyperintensity in the globus pallidus of CHS in patients, with higher diagnostic accuracy in differentiating MHE from non-MHI in CHS patients with a mean age of 70 years. Furthermore, emphasizing its significance over conventional psychometric tests as a non-invasive and objective tool for detecting MHE in CHS patients, and potentially serving as a surrogate biomarker for the progression of chronic liver diseases.

**Reviewer’s Comments and Suggestions**

1. Please define the acronym “hepatic encephalopathy (PH)” upon its first mention, and use it consistently thereafter.
2. “ PHES is considered the “gold” standard diagnosis for MHE, which includes five tests.”

The sentence requires reference, and it would be informative to list down the tests briefly, accompanied with a reference as follows: “ The PHES (psychometric hepatic encephalopathy score) test is deemed the gold standard in the diagnosis of MHE, consisting of 5 paper-and-pencil test sheets: Number connection tests (NCT-A and NCT-B), line tracing test (LTT), digit symbol test(DST), and serial dotting test (SDT).

3. The authors mentioned that the diagnosis of MHE relies on the results of two abnormal tests. Which two tests are you referring to? The EASL/AASL 2014 Guidelines on Hepatic Encephalopathy recommended using at least two tests to detect MHE, based on resulting alterations in psychometric and neurophysiologic tests. However, the updated EASL guidelines from 2022 recommend relying on a single test, with an emphasis on available local norms and experience. Can you give charity on this matter?
4. The authors highlighted two significant limitations of applying PHES tests for diagnosing MHE: the time-consuming nature of the tests and the influence of participants' educational level on outcomes. However, the authors fall short in addressing the age of study subjects as one of the prominent factors correlated with mental function, performance, and patient cognitive decline.
5. According to (Randolph, C. et al.), the Neuropsychological Assessment of Hepatic Encephalopathy: ISHEN’s 2009 Practice Guidelines pointed out that the PHES test findings are impacted by various factors, including age, gender, level of education, alcohol consumption, and visual abilities of the individual attending the psychometric test. Since the test focuses on multiple domains (processing speed, working memory, mental attention, visual scanning efficacy and perception, fine motor function, and motor speed), the age of the test subject, as well as occupation, social background, and gender, affect the sensitivity of the tests and shouldn’t be left unnoted as statistically significant parameters of performance.
6. Several scientific studies stated that neuronal loss and manganese accumulation due to hepatic encephalopathy can lead to bilateral, reversibly symmetrical T1 hyperintensity in the globus pallidus and substantia nigra areas of the brain.
7. Evidence from Magnetic resonance imaging has shown that T2 hyperintensity occurs in the white matter along the corticospinal tract in the brains of patients with advanced hepatic encephalopathy and cirrhosis. Encephalopathy (MHE) does not have a consistent or specific MRI T2 hyperintensity signature, as MHE is primarily a clinical diagnosis based on psychometric tests. What is the rationale behind including T2 mapping in your research study?
8. In your ethical considerations section, you mentioned the potential risks and benefits involved in the study. Are you referring to the risks and benefits of MRI examination? Or, the rewards and drawbacks of T1 and T2 mapping? Can you elaborate on it with one or two lines?
9. What are you implying by using the term forward-looking observational study? Was it a retrospective study with follow-up measurements of previously collected data from patients? Or a prospective longitudinal survey observing the two cohorts for a specific period of time?
10. The study design stated that the participants were divided into two respective groups (with MHI and without MHI), with a sample size of approximately 36 patients. However, your study flow diagram (Fig. 1) reveals a discrepancy in sample size between these two groups (52 vs. 36). Could you provide clarity on this matter?
11. Were the 50 patients with MHE and 38 patients without MHE (non-MHE) comparable groups in your study?
12. Your exclusion criteria were a history of cirrhosis, alcoholism, hepatocellular carcinoma, and medication usage. Did you have a patient's information on historical neuropsychological disorders, brain injury, and neurological diseases that potentially affect the cognitive function and bias the assessment of study subjects?
13. Was magnetic resonance imaging employed in the heart or brain regions? Can you provide us with the context for why your MRI examinations were specifically targeted to assess myocardial tissue characterization? Or, is it a typographic error that needs correction?
14. What normality test did you use to assess the distribution of the continuous variable?
15. Did you have any missing values and outliers in your variables? How did you handle it? What techniques were implemented during the data cleaning phases?
16. Fig. 3 shows the association between MRI and clinical features using a Pearson correlation matrix. Did you use the Spearman correlation matrix in your univariate analysis? Since you stated Pearson and Spearman correlation analyses in your statistical section, which variables were found to be non-normally distributed? Please specify the findings in your results section.
17. Multivariate adjustment is reported in your results section. Please specify all your analysis techniques clearly in the statistical section.
18. What variables were adjusted in your logistic regression? Define your dependent variable clearly, independent predictors, and adjusted co-founders in your model.
19. The authors aimed to evaluate the diagnostic accuracy of T1 and T2 mapping for detecting MHE. However, the diagnostic performance of T2 is missing in the results section. Did your T2 mapping comparison between groups show statistically non-significant results in all tests, including descriptive, univariate, multivariate, and ROC analysis? If that is the case, the scope of your research study should focus on only T1 mapping rather than T2.
20. N (%). Please specify it in the footnotes of Table 1 that your continuous variables are presented as the mean ± standard deviation, and categorical variables are presented as frequency values in numbers and percentages (%).
21. It would be informative if you could present multivariate analysis as Table 2 and ROC analysis as Fig. 3.
