# Supplementary material for: The role of quantitative T1 and T2 mapping for detecting minimal hepatic encephalopathy in chronic hepatic schistosomiasis patients
Source: PLoS One. 2026 Jan 6;21(1):e0339588. doi: 10.1371/journal.pone.0339588 (PMC12774341; doi:10.1371/journal.pone.0339588)
Supplement: Attachment — Submitted filename: Response to Reviewers.docx [file pone.0339588.s003.docx]

Thank you for your detailed review and constructive feedback. We've carefully addressed each comment with comprehensive revisions. Below is a point-by-point response, with changes highlighted in bold or tracked in the attached document for clarity.

Additional Editor Comments:

1) ABSTRACT:

 The abstract will be better if structured in this way: background, aim, methods, results, and conclusion.

Response: Thank you for the suggestion. We have structured the abstract as suggested.

 Methods is lacking the study setting.

Response:

Thank you for the comment. We have revised the Methods of the abstract to include the study setting.

2) INTRODUCTION:

 Newest Global/ Regional/ China prevalence of minimal hepatic encephalopathy among chronic hepatic schistosomiasis patients should be stated.

 The current situation of other developed and developing countries should also be added with new references.

 Explaining why this topic was chosen for analysis in this article is not well written. The benefits of conducting the study to the community should be explained.

Response: Thank you for the suggestions. We have revised the introduction to include updated prevalence data, situation of other developed and developing countries, and a clearer rationale for the study.

3) METHODS:

 How did the authors get the study subjects? They have to clearly address their sampling technique?

Response: Thank you for the comment. We have revised the manuscript to provide explicit details about our sampling technique (Materials and methods-Data acquisition).

 The study design was a cross-sectional observational study not a forward-looking observational study.

Response: Thank you for the reminder. This is a cross-sectional observational study. We corrected it in the text (Materials and methods-Study design and determination of sample size).

Methods is lacking the study setting.

 How the Authors got the socioeconomic data from the patients?

Response: Thank you for the comment. In this study, we did not collect detailed socioeconomic variables such as income or occupation. However, we recorded education level along with medical history and age, which were obtained through a structured questionnaire completed during patient enrollment, before neuropsychological testing (Materials and methods-Data acquisition).

 How the participants answered the questionnaire, by themselves or with assistance from researcher?

 How long did it take to complete each test/questionnaire?

 The authors should mention the criteria of the study setting.

 It is advisable to include the questions as per each domain in the methodology.

 How to score the NCT-A and the DST tests, specify the cut points?

Response: Thank you for the comment. We have added details regarding the test administration procedure, completion times for each assessment, details about the study setting (Materials and methods-Assessment of minimal hepatic encephalopathy).

 T1 and T2 mapping need more clarification of their indication and usage.

Response: Thank you for the comment. We have revised the manuscript to provide a clearer explanation of T1 and T2 mapping methodologies and their specific clinical applications (Materials and methods-Magnetic resonance imaging protocol).

 What is the difference between conventional T1-weighted imaging and quantitative T1 mapping?

Response: Thank you for the inquiry. Conventional T1-Weighted Imaging provides a qualitative method based on signal intensity, relying on radiologists' subjective interpretation. Quantitative T1 Mapping offers objective, reproducible assessment of T1 relaxation times.

 What strategy was devised to increase the accuracy of the study and the accuracy of the answers? What types of technique the authors used to keep/control the data quality?

Response: Thank you for the inquiry. All MRI examinations and psychometric tests followed standardized operating protocols. Two experienced radiologists, blinded to clinical data, independently measured T1 and T2 values, with discrepancies resolved by consensus. Images with artifacts were excluded from analysis. Psychometric tests were administered by trained examiners using uniform instructions. Data entry was performed in duplicate and cross-checked to minimize transcription errors.

 Authors should include a reference for using the stated formula in calculating the sample size. Furthermore, the basis of sample size calculation should be mentioned to know the confidence level and the margin of error. How did you determine the sample size? Please give a justification for the sample size.

Response: Thank you for the comment. We revised the text to clarify the sample size calculating method (Materials and methods-Study design and determination of sample size).

4) RESULTS:

 Number of the study participants in both groups in Figure 1. The work flow of this study was differed from what written in the result's comment.

Response: Thank you for the comment. We have included the number of the study participants and modified the work flow to be consistent with the text.

 The result section is lacking tables for NCT-A and DST scores, multivariable logistic regression, and ROC analysis figure and table.

Response: Thank you for the comment. The NCT-A and DST scores have been included in Table 1. The result of the multivariable logistic regression has been included in Supplementary Table 2. ROC analysis has been included in the Table 2.

 Multivariable logistic regression table should contain all variables enter in the regression.

Response: Thank you for the comment. We considered age, gender, and education level as potential confounding variables, along with the variables with p < 0.05 (AST, T1 frontal lobe, T1 temporal lobe, T1 occipital lobe, T1 caudate nucleus, and T1 globus pallidus) in univariate analyses. The result of the multivariable logistic regression analysis is now provided in the Supplementary Table 2.

5) DISCUSSION:

 The authors had not done a good job of connecting their findings to the literature and interpreting their findings. Discuss by using the scientific reasoning the MRI T1 and T2 mapping for detecting MHE among CHS patients in other developing and developed countries with similar context. The manuscript could be greatly strengthened if the authors could compare the findings of the study with other findings and state the reasons for the strengths and weaknesses in each section.

Response: Thank you for the suggestion. We revised the Discussion to highlight the strengths and limitations of our results relative to other studies.

6) STRENGTHS:

 Please analyze the strengths of the study.

Response: Thank you for the suggestion. We added a paragraph summarizing the key methodological and clinical strengths of this study.

Reviewer #1: Peer Review

Introduction

1. The introduction provides useful background but is somewhat lengthy and diffuse. The rationale could be sharpened to help the reader follow clearly what is new, what is relevant, and how MRI is currently used. At present, MRI findings such as T1 hyperintensities are research observations reflecting manganese deposition but are not part of routine diagnostic criteria for MHE and do not replace psychometric testing in clinical practice. Quantitative T1 mapping has shown promise in cirrhosis and animal models but has not previously been studied in CHS. Making this distinction more explicit would clarify the knowledge gap and highlight the novelty of the present study.

Response: Thank you for the suggestion. We have revised the introduction as suggested.

2. The final paragraph should concisely state the study's objective and hypothesis (e.g., that quantitative T1 mapping, especially in the globus pallidus, may discriminate between CHS patients with and without MHE).

Response: Thank you for the suggestion. We revised the introduction to make the study's objective and hypothesis clearer for the readers (Introduction-last paragraph).

Methods

3. The study design should be described as a cross-sectional observational study, rather than "forward-looking observational."

Response: Thank you for the reminder. This is a cross-sectional observational study. We corrected it in the text (Materials and methods-Study design and determination of sample size).

4. The sample size calculation is reported, which is a strength, but the assumptions are unclear. A reference or justification for the expected effect size should be provided, and it should be specified whether the same calculation applied to both T1 and T2 outcomes.

Response: Thank you for the comment. We revised the text to clarify the sample size calculating method (Materials and methods-Study design and determination of sample size).

5. Exclusion criteria include "history of cirrhosis." Since schistosomiasis patients may have periportal fibrosis or secondary portal hypertension, the operational definition of cirrhosis and how it was excluded should be clarified.

Response: Thank you for the comment. Cirrhosis was defined based on a combination of clinical history. Patients with a history of cirrhosis were excluded.

6. Diagnosis of MHE relied on NCT-A and DST. The authors should justify this choice, since PHES is considered the gold standard internationally. It would also be useful to discuss how much diagnostic overlap exists with PHES and whether using only two tests may reduce accuracy.

Response: Thank you for the comment. We have added text to clarify this justification and briefly discuss potential diagnostic limitations (Discussion-last paragraph).

7. The MRI protocol is described in detail, but further clarification is needed: were regions analyzed automatically or manually? If manual, how many raters were involved, and was inter-rater reliability (e.g., ICC) calculated? Were image readers blinded to test results or group allocation?

Response: Thank you for the comment. Quantitative T1 and T2 values were obtained by manually delineating regions of interest (ROIs) in the frontal, temporal, and occipital lobes, caudate nucleus, and globus pallidus. Two experienced radiologists, blinded to clinical data, independently measured T1 and T2 values, with discrepancies resolved by consensus. The mean of the two measurements was used for statistical analysis (Materials and methods-Magnetic resonance imaging protocol). Although the inter-rater reliability (ICC) was not calculated, both radiologists followed a standardized ROI placement protocol to ensure consistency across measurements.

8. Blinding of neuropsychological test assessors should also be stated.

Response: Thank you for the comment. We mentioned it in the text (Materials and methods-Magnetic resonance imaging protocol).

9. The regression models are not fully transparent. The covariates included should be explicitly listed, with justification for their selection. Handling of multiple comparisons should also be mentioned.

Response: Thank you for the comment. We considered age, gender, and education level as potential confounding variables, along with the variables with p < 0.05 (AST, T1 frontal lobe, T1 temporal lobe, T1 occipital lobe, T1 caudate nucleus, and T1 globus pallidus) in univariate analyses. The result of the multivariable logistic regression analysis is now provided in the Supplementary Table 2.

10. Figure 1 illustrates the study workflow but does not report the number of patients screened, excluded, and included. For clarity and compliance with reporting standards, these numbers should be added.

Response: Thank you for the comment. We have included the number of the study participants and modified the work flow to be consistent with the text.

Results

11. The results are clearly presented, but Table 1 contains many non-significant variables that could be moved to supplementary material or presented more concisely.

Response: Thank you for the suggestion. We have moved the non-significant variables to the Supplementary Table 1.

12. Confidence intervals should be consistently reported alongside p-values to improve interpretability.

Response: Thank you for the suggestion. We have revised the manuscript to include 95% confidence intervals for mean differences, odds ratios, and other relevant estimates in the Results section and in Supplementary Table 1.

13. The covariates used in regression should be specified in both methods and results to increase transparency.

Response: Thank you for the suggestion. We have added the methods and results of the multivariable logistic regression analysis in the text (Statistical Analysis and Multivariable adjustment and correlation analyses).

Discussion

14. The very high AUC values (0.92 with sensitivity 0.97 and specificity 0.90) should be interpreted cautiously. Possible sources of overestimation, such as single-center recruitment, sample size, or limited cohort variability, should be discussed.

Response: Thank you for the suggestion. We have revised the manuscript to discuss these limitations (Discussion-last paragraph).

15. The statement that T1 mapping is "more objective and reproducible" than conventional T1-weighted imaging should be supported with references or rephrased more cautiously.

Response: Thank you for the suggestion. We have revised the manuscript with more appropriate language (Conclusions).

16. The limitations of psychometric testing related to education are important, but no socioeconomic or educational baseline variables are reported. If these data are unavailable, this should be acknowledged as a limitation.

Response: Thank you for the suggestion. We added the information about educational level in the text.

17. Clinical implications should be framed carefully. At present, T1 mapping should be considered as a complementary tool rather than a replacement for psychometric testing. Barriers such as availability and cost should also be acknowledged.

Response: Thank you for the suggestion. We have revised the conclusion and discussion as suggested.

Conclusion

18. The conclusion is consistent with the findings but should avoid overstatement. Instead of claiming "strong evidence," the study should be described as providing promising evidence that T1 mapping has potential as a biomarker for MHE in CHS.

Response: Thank you for the suggestion. We revised the conclusion in both the abstract and the main text to avoid overstatement about the clinical usefulness of quantitative T1 mapping.

Reviewer #2: Well written and articulated study. If you can elaborate if similar T1 and T2 findings seen in cirrhosis related MHE or if this is different readers will have a better understanding.

Response: Thank you for the suggestion. We have revised the manuscript to include a comparative discussion of these findings (Discussion).

How were the candidates selected for the scan? If it was every single patient presenting to the clinic with CHS or was there a minimal severity required?

Response: Thank you for the inquiry. In our study, consecutive CHS patients referred to the local hepatology clinics and the gastroenterology department were screened for eligibility. All patients were invited to participate if they had a confirmed diagnosis of CHS and were willing and able to undergo MRI examination and psychometric testing. The MHE status was determined after enrollment based on psychometric test results (NCT-A and DST), allowing classification into MHE and non-MHE groups for comparative analysis.

Reviewer #3: Strengths

Minor Suggestions for Revision

Consistency in terms: Ensure consistent use of "CHS" vs. "CHE" (one instance of "CHE" appears in the Results section).

Response: Thank you for the suggestion. We revised CHE to CHS for consistency throughout the text.

Reviewer #4:

Reviewer's Comments and Suggestions

Please define the acronym "hepatic encephalopathy (PH)" upon its first mention, and use it consistently thereafter.

Response: Thank you for the suggestion. We have used "HE" as the acronym for hepatic encephalopathy and used it consistently throughout the text.

" PHES is considered the "gold" standard diagnosis for MHE, which includes five tests."

The sentence requires reference, and it would be informative to list down the tests briefly, accompanied with a reference as follows: " The PHES (psychometric hepatic encephalopathy score) test is deemed the gold standard in the diagnosis of MHE, consisting of 5 paper-and-pencil test sheets: Number connection tests (NCT-A and NCT-B), line tracing test (LTT), digit symbol test(DST), and serial dotting test (SDT).

Response: Thank you for the suggestion. We have listed all these five tests with references (Introduction).

The authors mentioned that the diagnosis of MHE relies on the results of two abnormal tests. Which two tests are you referring to? The EASL/AASL 2014 Guidelines on Hepatic Encephalopathy recommended using at least two tests to detect MHE, based on resulting alterations in psychometric and neurophysiologic tests. However, the updated EASL guidelines from 2022 recommend relying on a single test, with an emphasis on available local norms and experience. Can you give charity on this matter?

Response: Thank you for the comment. The 2022 EASL guidelines have since updated recommendations to allow single-test diagnosis when locally validated norms are available, our two-test approach may provide robust diagnostic certainty.

The authors highlighted two significant limitations of applying PHES tests for diagnosing MHE: the time-consuming nature of the tests and the influence of participants' educational level on outcomes. However, the authors fall short in addressing the age of study subjects as one of the prominent factors correlated with mental function, performance, and patient cognitive decline.

According to (Randolph, C. et al.), the Neuropsychological Assessment of Hepatic Encephalopathy: ISHEN's 2009 Practice Guidelines pointed out that the PHES test findings are impacted by various factors, including age, gender, level of education, alcohol consumption, and visual abilities of the individual attending the psychometric test. Since the test focuses on multiple domains (processing speed, working memory, mental attention, visual scanning efficacy and perception, fine motor function, and motor speed), the age of the test subject, as well as occupation, social background, and gender, affect the sensitivity of the tests and shouldn't be left unnoted as statistically significant parameters of performance.

**Response:** Thank you for the comment. We considered age, gender, and education level as potential confounding variables, along with the variables with p < 0.05 (AST, T1 frontal lobe, T1 temporal lobe, T1 occipital lobe, T1 caudate nucleus, and T1 globus pallidus) in univariate analyses. Multivariable logistic regression analysis revealed that none of the potential confounders (age, gender, education level, AST, T1 Frontal Lobe, T1 Temporal Lobe, T1 Occipital Lobe, and T1 Caudate Nucleus) were independently associated with MHE after adjustment (all p > 0.05). Lower T1 value in globus pallidus was strongly associated with an increased likelihood of MHE. We added it in the Statistical Analysis section. And the result of the multivariable logistic regression analysis is now provided in the Supplementary Table 2. We also revised the text to address age as a critical confounding factor in psychometric assessment as a limitation of this study (Discussion-last paragraph).

Several scientific studies stated that neuronal loss and manganese accumulation due to hepatic encephalopathy can lead to bilateral, reversibly symmetrical T1 hyperintensity in the globus pallidus and substantia nigra areas of the brain.

Response: Thank you for the comment. The bilateral, symmetrical T1 hyperintensity in the globus pallidus and substantia nigra is the main finding in hepatic encephalopathy, resulting from manganese accumulation due to portosystemic shunting and bypassing of hepatic metabolism. Our quantitative T1 mapping findings are consistent with this phenomenon, as the lower T1 values (T1 shortening) we observed correspond to the T1 hyperintensity seen on conventional T1-weighted imaging.

Evidence from Magnetic resonance imaging has shown that T2 hyperintensity occurs in the white matter along the corticospinal tract in the brains of patients with advanced hepatic encephalopathy and cirrhosis. Encephalopathy (MHE) does not have a consistent or specific MRI T2 hyperintensity signature, as MHE is primarily a clinical diagnosis based on psychometric tests. What is the rationale behind including T2 mapping in your research study?

**Response:** Thank you for the comment. Quantitative T2 mapping was included as an exploratory component in our study to assess whether subtle microstructural or water-content changes, undetectable on standard T2-weighted imaging, could provide additional information about early cerebral alterations in CHS-related MHE. While our findings confirmed that T2 values did not differ significantly between MHE and non-MHE patients.

In your ethical considerations section, you mentioned the potential risks and benefits involved in the study. Are you referring to the risks and benefits of MRI examination? Or, the rewards and drawbacks of T1 and T2 mapping? Can you elaborate on it with one or two lines?

Response: Thank you for the inquiry. We referred to the risks and benefits associated with the MRI examination itself. We have revised the expression for clarity (Materials and methods-Ethical considerations).

What are you implying by using the term forward-looking observational study? Was it a retrospective study with follow-up measurements of previously collected data from patients? Or a prospective longitudinal survey observing the two cohorts for a specific period of time?

Response: Thank you for the reminder. This is a cross-sectional observational study. We corrected it in the text (Materials and methods-Study design and determination of sample size).

The study design stated that the participants were divided into two respective groups (with MHI and without MHI), with a sample size of approximately 36 patients. However, your study flow diagram (Fig. 1) reveals a discrepancy in sample size between these two groups (52 vs. 36). Could you provide clarity on this matter?

Were the 50 patients with MHE and 38 patients without MHE (non-MHE) comparable groups in your study?

Response: Thank you for the inquiry. The sample size of 36 per group referred to the minimum estimated number required from the power calculation, not the final number of enrolled participants. In practice, 88 eligible patients were included (50 with MHE and 38 without MHE) (Materials and methods-Study design and determination of sample size).

Your exclusion criteria were a history of cirrhosis, alcoholism, hepatocellular carcinoma, and medication usage. Did you have a patient's information on historical neuropsychological disorders, brain injury, and neurological diseases that potentially affect the cognitive function and bias the assessment of study subjects?

Response: Thank you for the inquiry. Before recruiting subjects for the study, we specifically inquired about the medical histories mentioned above. Patients with such conditions were also excluded from the study (Materials and methods-Data acquisition).

Was magnetic resonance imaging employed in the heart or brain regions? Can you provide us with the context for why your MRI examinations were specifically targeted to assess myocardial tissue characterization? Or, is it a typographic error that needs correction?

Response: Thank you for the inquiry. The MRI was equipped with advanced quantitative mapping capabilities to assess the comprehensive characterization of brain regions. We corrected this error in the text (Materials and methods-Magnetic resonance imaging protocol).

What normality test did you use to assess the distribution of the continuous variable?

Did you have any missing values and outliers in your variables? How did you handle it? What techniques were implemented during the data cleaning phases?

Response: Thank you for the inquiry. Normality of all continuous variables was evaluated using the Shapiro-Wilk test. No variable had more than 5% missing data. Missing data of laboratory or MRI values and outliers were excluded. During data cleaning, we conducted cross-checks for data consistency, verified range validity, standardized variable units, and ensured anonymization.

Fig. 3 shows the association between MRI and clinical features using a Pearson correlation matrix. Did you use the Spearman correlation matrix in your univariate analysis? Since you stated Pearson and Spearman correlation analyses in your statistical section, which variables were found to be non-normally distributed? Please specify the findings in your results section.

Response: Thank you for the suggestion. Normality of all continuous variables was evaluated using the Shapiro-Wilk test. Most MRI-derived T1 and T2 values, neuropsychological test scores (NCT-A and DST), and ALB followed a normal distribution; therefore, Pearson correlation was used for these analyses. However, certain liver function variables (AST, ALT, TB, PT, INR and platelet count) were not normally distributed. Thus, Spearman correlation was used when these variables were included in exploratory analyses. We clarified this in the text (Materials and methods-Multivariable adjustment and correlation analyses).

Multivariate adjustment is reported in your results section. Please specify all your analysis techniques clearly in the statistical section.

Response: Thank you for the suggestion. We have now revised the text to clearly describe all statistical techniques used (Materials and methods-Statistical analysis).

What variables were adjusted in your logistic regression? Define your dependent variable clearly, independent predictors, and adjusted co-founders in your model.

Response: Thank you for the inquiry. We considered age, gender, and education level as potential confounding variables, along with the variables with p < 0.05 (AST, T1 frontal lobe, T1 temporal lobe, T1 occipital lobe, T1 caudate nucleus, and T1 globus pallidus) in univariate analyses. Multivariable logistic regression analysis revealed that none of the potential confounders (age, gender, education level, AST, T1 Frontal Lobe, T1 Temporal Lobe, T1 Occipital Lobe, and T1 Caudate Nucleus) were independently associated with MHE after adjustment (all p > 0.05). Lower T1 value in globus pallidus was strongly associated with an increased likelihood of MHE. We added it in the Statistical Analysis section. And the result of the multivariable logistic regression analysis is now provided in the Supplementary Table 2.

The authors aimed to evaluate the diagnostic accuracy of T1 and T2 mapping for detecting MHE. However, the diagnostic performance of T2 is missing in the results section. Did your T2 mapping comparison between groups show statistically non-significant results in all tests, including descriptive, univariate, multivariate, and ROC analysis? If that is the case, the scope of your research study should focus on only T1 mapping rather than T2.

Response: Thank you for the suggestion. Indeed, the T2 mapping results did not demonstrate significant differences between MHE and non-MHE groups. Therefore, we have clarified these results in the abstract, results, and discussion.

N (%). Please specify it in the footnotes of Table 1 that your continuous variables are presented as the mean ± standard deviation, and categorical variables are presented as frequency values in numbers and percentages (%).

Response: Thank you for the suggestion. We added a footnote below Table 1 as suggested.

It would be informative if you could present multivariate analysis as Table 2 and ROC analysis as Fig. 3.

Response: Thank you for the suggestion. We have added a new Supplementary Table 2 to summarize the results of the multivariable logistic regression analysis and a new Table 2 to illustrate the ROC curve analysis for T1 values in distinguishing MHE from non-MHE patients.

Reviewer #5: Methods and Results

Did you identify any confounders in this study?

What parameters were adjusted for in this study?

Include results of the sensitivity analysis done to ensure robustness of the data presented

Response: Thank you for the inquiry. We considered age, gender, and education level as potential confounding variables, along with the variables with p < 0.05 (AST, T1 frontal lobe, T1 temporal lobe, T1 occipital lobe, T1 caudate nucleus, and T1 globus pallidus) in univariate analyses. Multivariable logistic regression analysis revealed that none of the potential confounders (age, gender, education level, AST, T1 Frontal Lobe, T1 Temporal Lobe, T1 Occipital Lobe, and T1 Caudate Nucleus) were independently associated with MHE after adjustment (all p > 0.05). Lower T1 value in globus pallidus was strongly associated with an increased likelihood of MHE. We added it in the Statistical Analysis section. And the result of the multivariable logistic regression analysis is now provided in the Supplementary Table 2.
