# Supplementary material for: The role of quantitative T1 and T2 mapping for detecting minimal hepatic encephalopathy in chronic hepatic schistosomiasis patients
Source: PLoS One. 2026 Jan 6;21(1):e0339588. doi: 10.1371/journal.pone.0339588 (PMC12774341; doi:10.1371/journal.pone.0339588)
Supplement: Attachment — Submitted filename: Response_to_Reviewers_auresp_2.docx [file pone.0339588.s004.docx]

Thank you for your meticulous review and insightful feedback. We have carefully reviewed each comment and implemented comprehensive revisions accordingly. A detailed point-by-point response is provided below, with all modifications tracked in the text for your convenience.

Reviewer #1

Remaining minor points for clarification

1. Blinding procedures

The revised text specifies that two radiologists independently analyzed MRI data blinded to clinical information, which is commendable. However, it remains unclear whether they were also blinded to participants' MHE classification status. I suppose they were, but this could be explicitly stated if it was the case. Similarly, while the psychometric tests were administered using standardized instructions by trained examiners, it is not explicitly stated whether these examiners were aware of the patients' cognitive or clinical background. Clarifying these aspects, whether both MRI readers and psychometric assessors were fully blinded to group status, would help the reader better assess the internal validity of the findings and minimize concerns about potential measurement bias.

Response: Thank you for the comment. In our study, both MRI readers and psychometric test examiners were fully blinded to the participants’ MHE classification status and clinical information. We have clarified it in the text.

2. Education variable

Including education level in Table 1 is an important improvement, but the variable ("above/below primary school") remains rather coarse. Education is known to influence psychometric performance through factors such as literacy, occupational complexity, and cognitive stimulation, which may not be captured by this binary measure. The discussion could benefit from a short acknowledgment that this simplified categorization may not fully account for the effect of educational background on test performance, particularly in older adults.

Response: Thank you for the comment. We have added a statement in the Discussion section acknowledging this limitation.

3. Clinical applicability and interpretation of diagnostic metrics

The study convincingly demonstrates that quantitative T1 mapping discriminates between CHS patients with and without MHE. However, as MHE status was determined prior to MRI, the reported AUC, sensitivity, specificity, PPV, and NPV describe discrimination within a predefined cohort rather than diagnostic accuracy in a real-world clinical population where MHE is suspected but not yet classified. The authors may wish to clarify this distinction in the Discussion to avoid overinterpretation. The current findings provide evidence of biomarker potential and pathophysiological relevance but do not yet establish clinical diagnostic performance.

Response: Thank you for the comment. We have revised the Discussion to clarify the distinction between biomarker discrimination and clinical diagnostic validation. The new text highlights that further prospective validation in unclassified patient populations is needed to confirm real-world diagnostic utility.

4. Power analysis

The revised version now specifies that the sample size calculation was performed post hoc based on the study results. This clarification is appreciated, but the authors could consider noting this explicitly as a limitation in the Discussion. A post hoc calculation does not inform study design or recruitment targets and therefore provides a retrospective estimate of statistical power rather than evidence of adequate pre-study planning. A short acknowledgment of this would improve transparency.

Response: Thank you for the comment. We have revised the Discussion section to note this limitation, emphasizing that our post hoc analysis aimed to assess achieved power rather than guide sample size determination prospectively.

Reviewer #4

Comment #1, “ The study design should be corrected with cross-sectional observational study, since the data is collected at a single point of time.”

Response: Thank you for the comment. Accordingly, we have corrected the terminology throughout the manuscript to ensure consistency and accuracy.

Comment #2, “ Ensure that all the key words are stated in the abstract section including T2, NCT-A, DST, globus pallidus and China.”

Response: Thank you for the comment. We have revised the Abstract to ensure that all the key terms “T2, NCT-A, DST, globus pallidus, and China” are explicitly mentioned.

Comment #3, “ The manuscript lacks background information and review of previous work on T2 mapping. It should be stated briefly in one or two lines.

Response: Thank you for the comment. We have revised the Introduction section to include a short statement summarizing the role and prior findings of T2 mapping in hepatic encephalopathy research.

Comment #4, “ Please include an operational definition of variables in your method section. Define,(Type B MHI,minimal hepatic encephalopathy, overt HE, and chronic CHS.)

Response: Thank you for the comment. We have revised the Methods section to explicitly define Type B MHE, minimal hepatic encephalopathy (MHE), overt hepatic encephalopathy (HE), and chronic hepatic schistosomiasis (CHS), based on established diagnostic criteria and previous literature.

Comment #5, “ Since tables and figures reflect your major findings, it will be better if you can present them in the result section rather than supplementary materials.

Response: Thank you for the comment. We kept some of the tables in supplementary materials to maintain main text flow, with detailed data there for interested readers.

Comment #6, “ Please put a brief emphasis in your discussion that the findings for your research outcome is generated from.middle age and older CHS patients. ( N.B. The mean age of the patients in your study was approximately 70 and most of them were in their 5th, 6th and 7th decades of life.)

Response: Thank you for the comment. We have added a sentence in the Discussion highlighting that our findings may be most relevant to this demographic and that age-specific variations should be explored in future research.

Comment #7, “ As the concluding statement of your discussion, you should highlight the quantitative role and diagnostic accuracy of T1 mapping in diagnosing MHE in middle aged and older CHS patients and its implications in the general population.

Response: Thank you for the comment. We have revised the conclusion section to better highlight the quantitative diagnostic role of T1 mapping and clarify its implications for the general population.

Comment #8, “ Make sure you state the key Messages of the scientific study in your conclusion section by raising the key points of MRI characterization; (a) T1 hyperintensity in the globus pallidus of CHS patients highlighting its diagnostic accuracy in differentiating MHE from non-MHI in CHS patients with mean age of 70, (b) emphasize its significance over conventional psychometric tests as a promising non-invasive and objective tool for detecting MHI in CHS patients and as potential surrogate marker for indicating the progression of chronic

Response: Thank you for the comment. We have substantially revised the conclusion section to clearly articulate the main findings as suggested.
