# Supplementary material for: The role of quantitative T1 and T2 mapping for detecting minimal hepatic encephalopathy in chronic hepatic schistosomiasis patients
Source: PLoS One. 2026 Jan 6;21(1):e0339588. doi: 10.1371/journal.pone.0339588 (PMC12774341; doi:10.1371/journal.pone.0339588)
Supplement: Attachment — Submitted filename: Response to Reviewers_R3.docx [file pone.0339588.s005.docx]

Thank you for your comments. We have carefully reviewed each comment and implemented comprehensive revisions accordingly. A detailed point-by-point response is provided below, with all modifications tracked in the text.

Additional Editor Comments:
It is advisable to add the figure of the ROC curve analysis and the table of multivariable logistic regression in the result section rather than the supplementary materials.

Response: Thank you for the comment. We have added a figure of the ROC curve analysis. And the table of multivariable logistic regression is now add in the result section.
